# DASMiner: discovering and integrating data from DAS sources

**DOI:** 10.1186/1752-0509-3-109

**Published:** 2009-11-17

**Authors:** Diogo FT Veiga, Helena F Deus, Caner Akdemir, Ana Tereza R Vasconcelos, Jonas S Almeida

**Affiliations:** 1 Department of Bioinformatics and Computational Biology, The University of Texas MD Anderson Cancer Center, 1515 Holcombe Blvd Houston, TX 77030, USA; 2 Bioinformatics Laboratory, Laboratório Nacional de Computação Científica, Av Getúlio Vargas, 333 Petrópolis, Rio de Janeiro, Brazil

## Abstract

**Background:**

DAS is a widely adopted protocol for providing syntactic interoperability among biological databases. The popularity of DAS is due to a simplified and elegant mechanism for data exchange that consists of sources exposing their RESTful interfaces for data access. As a growing number of DAS services are available for molecular biology resources, there is an incentive to explore this protocol in order to advance data discovery and integration among these resources.

**Results:**

We developed DASMiner, a Matlab toolkit for querying DAS data sources that enables creation of integrated biological models using the information available in DAS-compliant repositories. DASMiner is composed by a browser application and an API that work together to facilitate gathering of data from different DAS sources, which can be used for creating enriched datasets from multiple sources.

The browser is used to formulate queries and navigate data contained in DAS sources. Users can execute queries against these sources in an intuitive fashion, without the need of knowing the specific DAS syntax for the particular source. Using the source's metadata provided by the DAS Registry, the browser's layout adapts to expose only the set of commands and coordinate systems supported by the specific source. For this reason, the browser can interrogate any DAS source, independently of the type of data being served.

The API component of DASMiner may be used for programmatic access of DAS sources by programs in Matlab. Once the desired data is found during navigation, the query is exported in the format of an API call to be used within any Matlab application. We illustrate the use of DASMiner by creating integrative models of histone modification maps and protein-protein interaction networks. These enriched datasets were built by retrieving and integrating distributed genomic and proteomic DAS sources using the API.

**Conclusion:**

The support of the DAS protocol allows that hundreds of molecular biology databases to be treated as a federated, online collection of resources. DASMiner enables full exploration of these resources, and can be used to deploy applications and create integrated views of biological systems using the information deposited in DAS repositories.

## Background

The DAS (Distributed Annotated System) was introduced in 2001 [1] as an integration mechanism primarily for sharing biological annotation of genome assemblies. Since then, a growing number of databases have been adopting the protocol to address the issue of aggregating data from external databases. Presently, more than 500 DAS sources, covering 40 species that publicly offer not only genomic annotations, but also a myriad of other data types, including protein records, protein-protein interactions, and transcriptomics data [2-5]. Thus, a large community of federated databases that share a domain of discourse in DAS has been established and can therefore be integrated. Furthermore, the DAS Registry was launched as a web-service that allows publishing and finding of DAS services [6]. The DAS Registry is keeping track of available DAS sources, providing metadata about them and monitoring the reliability of the service.

Presently, much open-source infrastructure has already been developed for DAS. On the server-side, implementations have been developed in Perl, as in the case of Proserver [7], as well as Java, namely myDAs [6] and Dazzle [8]. On the client-side, libraries for manipulating DAS data are available in Perl as Bio::Das::Lite [9] and in Java as Dasobert [10]. Also, DAS viewers such as Dasty2 [11], developed using Javascript and AJAX, or SPICE [12], using Java webstart technology, can be used to bundle and integrate data at the visualization level.

In this work we introduce DASMiner, a generic DAS browser and API designed for exploring both the DAS Registry as well as specific DAS sources. The motivation to develop the tool was twofold. First, we intended to facilitate access to DAS data for all users, not only for specialized DAS clients as genome browsers. Thus, the browser automates the process of writing DAS queries, making it transparent to the user. Second, we intended to make use of the DAS-stored experimental data to create integrative biological models. We demonstrate how to assemble enriched data sets of histone modification and molecular interaction data by accessing different DAS sources using the API. Then we also show how to create new visualizations for these integrated data sets.

## Implementation

**Figure 1:**
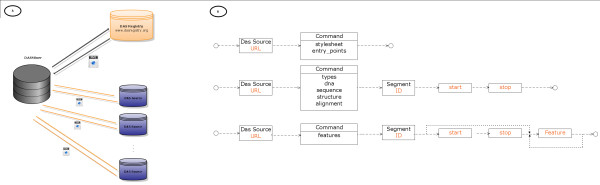
**Overview of the application architecture and DAS 1.53E protocol syntax commands**. (A) DASMiner communicates with the DAS Registry and with any DAS source. Interaction with the Registry is useful for finding new services as well as for retrieving sources metadata needed for configuring future queries against a specific source. (B) DAS 1.53E (extended) commands can be grouped in three categories, according to the kind of parameters they require. Here we are simplifying the syntax of the commands *alignment *and *interaction*, which can take other arguments. For complete descriptions, consult the latest version of DAS specification [22] and DAS Registry [23] specifications.

All components of DASMiner, namely the browser (GUI application) and the DAS API were written in Matlab, and are freely available for download (see Availability). An outline of the implemented software architecture is shown in Figure 1A. DASMiner interacts with the DAS Registry in order to retrieve metadata about a DAS service. The search for services (black arrow) is made through the DAS-style command *sources*, and the search can be refined by organism, coordinate systems, label, and capabilities (commands). The Registry returns metadata in response, in the format of an XML document, which includes the server URL as well as information about the coordinate systems and commands supported by the source. DASMiner uses this service metadata for customization and tuning of the GUI for browsing DAS sources. For example, if the source is providing annotation for a DNA segment such as UCSC Encode, the GUI opens the required fields chromosome, start, and stop. On the other hand, if the source requires the input of an Entrez ID or an Ensembl ID, such as Ensembl Human Genes, only an ID field is shown on the GUI.

### DASMiner Application Programming Interface (API)

The flexibility of the GUI relies on a correspondingly simple library of commands that supports the intricate workings of the DAS abstraction. The API is responsible for building and executing DAS queries or commands. Basically, the library has functions for connecting and reading DAS URLs, as well as for handling service exceptions such as badly formed XML and server timeout. Also, the core functions are dedicated to assembling DAS commands with the proper syntax. We identified three categories of commands in the version 1.53E protocol, as illustrated in Figure 1B. This diagram illustrates how the functions envelop a DAS request in a valid URL, by concatenating a series of elements and argument-value pairs. Then according to Figure 1B, the *entry_points *query is made up of the DAS server address and the command *entry_points *with no arguments. For instance, the URL http://www.ebi.ac.uk/das-srv/uniprot/das/uniprot/entry_points may used to retrieve the *entry_points *in the Uniprot DAS server. The equivalent API call to issue this query is made by specifying a series of attribute-value pairs of the *executeDASCommand *function, namely the URL of the service, the *command *and the *timeout *as in *executeDASCommand('http://www.ebi.ac.uk/das-srv/uniprot/das','command','dsn','timeout', 15000)*. The more elaborated command is *features*, used for retrieving annotation over a biological entity. *Features *requires as input the segment definition, and optionally the enumeration of desired features (Figure 1B). For example, the query to find out all SNPs and predicted genes in the chromosome 4, positions 3M-4M available at UCSC Genome database, the query is http://genome.ucsc.edu/cgi-bin/das/hg16/features?segment=4:3000000,4000000;type=snp;type=knownGene. The equivalent API call is the following: *executeDASCommand('http://genome.ucsc.edu/cgi-bin/das','command','features', 'DSN', 'hg16', 'chrom','4','start','3000000', 'stop', '4000000', 'featuresList', {'snp','knownGene'})*.

All functions in the API are thoroughly documented, with many examples of usage, and the documentation is available online at the accompanying website.

## Results

### Browser utilization

**Figure 2:**
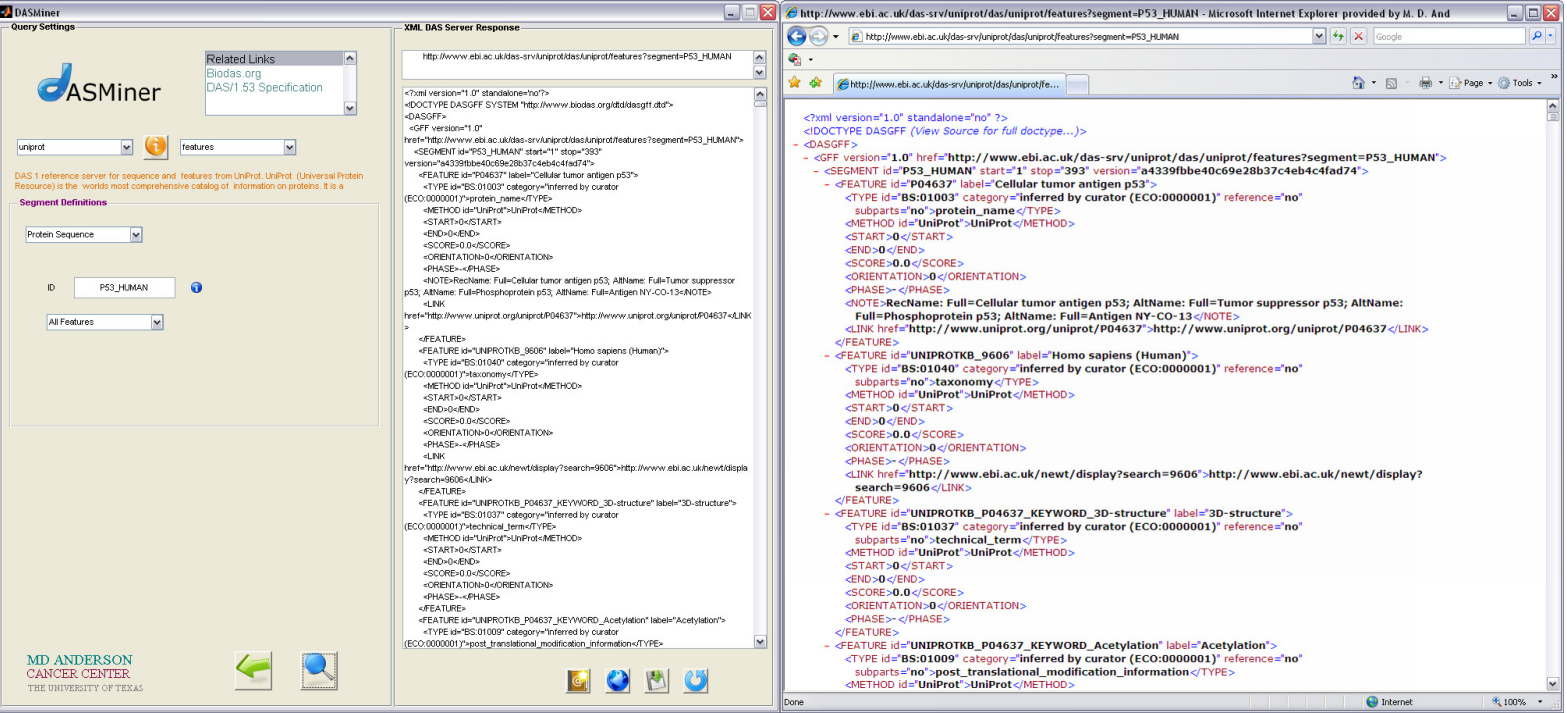
**Screenshot of the DASMiner interface and browser visualization of the XML response**. From left to right: (i) query settings panel, mainly showing the parameters used in the query (i.e. retrieving all features of the P53 human protein deposited in Uniprot); (ii) the display panel, which outputs the XML server response; and (iii) the XML output can be saved in a file or exported to the browser, as depicted.

DASMiner establishes a general purpose procedure for discovering and getting data from DAS sources. It explores the DAS formalism (Figure 1B) and provides an intuitive interface (Figure 2) without exposing the user to the minutia of the DAS commands syntax. Specifically, the application automates the process of writing DAS queries and allows the user to completely explore any DAS source by trying different commands and configurations; these are all explicitly available as alternative operations in the browser. The navigation of DAS sources is aided by *info *links pointing to the DAS Registry, which provides information about the service and hints on what type of input is expected (e.g. what kind of ID or coordinate system is accepted by the source). Since each DAS source can choose to implement a particular abstraction of the DAS protocol, i.e. a specific set of commands and coordinate system, the browser's layout changes to expose the set of commands and coordinate systems supported by the specific service.

The browser has two main panels (Figure 2): (i) a query definition panel, where the user chooses commands and sets their arguments, and (ii) a data display and export panel aimed at visualizing and manipulating the XML response from the DAS source.

The procedure of assembling a query was designed such that the user will be prompted to enter query settings in a cascade model. Depending on which command has been selected, fields will be displayed in the query settings panel, where parameters for the command should be typed. For instance, Figure 2 depicts how the browser will appear to the user running the *features *command to retrieve the annotation of the p53 Human protein from Uniprot. The first step to perform the query is to select the data source from the sources menu (Figure 2, upper left corner), which populates the interface with the info orange icon, the description of the source and the capabilities menu. The next step is to select *features *from the command menu, which results in the display of the segment definitions panel. This navigation follows the DAS generic model as described in the diagram in Figure 1B. Finally, the protein ID (*P53_HUMAN*) is provided, followed by the selection of 'All features' (default) or 'Browse features' in the feature selector menu and pushing the search button. The DAS request is then sent to the Uniprot DAS server, which will send back an XML-formatted response. All query information is saved as variables in the Matlab workspace, so that the user can manipulate query results easily. For example, after a query is performed, the user is informed that four variables are created in the workspace: (*i*) *DASquery_XML*: string, returned by DAS service; (*ii) DASquery_url*: string, URL assembled by the API to retrieve the data; (*iii*) *DASquery_struct*: struct, XML is transformed into a Matlab struct that can be explored (in the case studies below we used structs to manipulate DAS data); (*iv*) *DASquery_struct2*: struct, XML is transformed into a Matlab struct using an alternative parser that creates a DOM tree out of the XML string.

Additionally, the XML output can be either exported to a file or visualized in the browser (Figure 2). Also, the query URL (top data display panel) can be exported to the Matlab workspace in the form of an API call and can be either executed by the function *eval *or inserted into any script.

**Figure 3:**
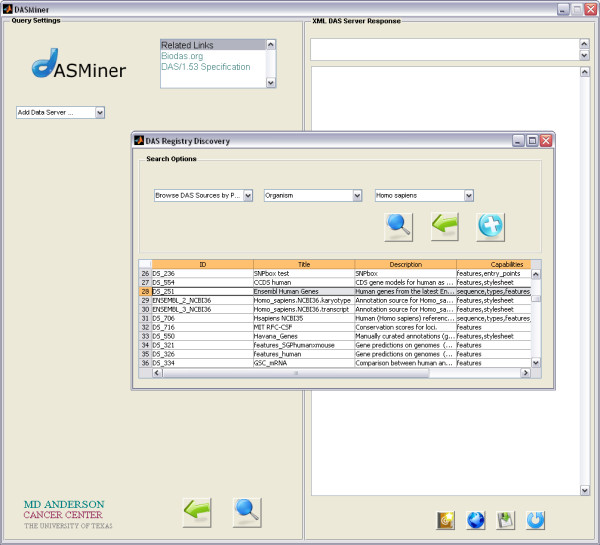
**Screenshot of the DASMiner search module for interacting with the DAS Registry**. The DAS Registry Discovery interface appears when the option 'Add Data Server' is selected in the Servers pull-down menu (very first menu in the left top corner). A search in the registry can be performed with different criteria, including by organism. The search results appear in a table, where each line refers to a DAS service matching the criterion. In this example, 57 DAS sources were found providing data about *Homo sapiens*.

A DAS Registry Discovery module has also been included to search the registry for sources (Figure 3). New services can be made available locally for browsing after being discovered through this module. The criteria for searching the registry include organism, coordinate system, authority, capability and label. As a criterion is selected, a pull-down menu is dynamically populated with the available options.

Figure 3 depicts the 57 DAS sources automatically retrieved for a query on *Homo sapiens*. The results table displays basic information about the sources, such as title, description and a link for the registry. The user can then select a DAS source for querying with the main interface.

### Examples of API applications

DASMiner API was used to create enriched data sets of histone modification data and protein interactions by accessing multiple DAS sources. The following case studies can be reproduced by running the files available in the Examples folder of the distributed source code. The example files are named by their correspondent Figure as described in the Figure captions. In general, the scripts for the examples execute DASMiner API calls to collect the data, parse the data locally to construct an appropriate data representation, and then plot a graphical visualization.

### A) Creating and visualizing enriched histone modification data sets

**Figure 4:**
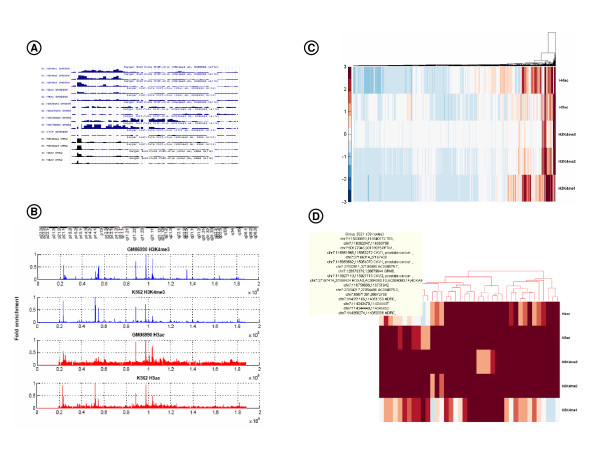
**Approaches for visualizing histone modification data**. A) The UCSC Genome browser provides area charts organized by cell lines. B) Comparison of H3K4me3 and H3ac histone signals in a normal (GM06990) and cancer (K562) cell lines, over chromosome 7. Data was retrieved from the UCSC DAS server using DASMiner API, and the plot can be easily prepared using *plottools*, a GUI tool to make graphics in Matlab (see file *Fig4B_histonesPlots_CancervsNormal.fig *in the examples folder of the distributed source code). C) Hierarchical clustering of histone profiles for DNA regions measured in chromosome 7, GM06990 cell line. The data set for clustering was built by fetching data from 3 DAS sources, namely ChIP-chip arrays from UCSC, gene annotation from Havana Database and cancer link from Genetic Association Disease. D) Group of 39 regions exhibiting strong enrichment of positive histone marks *H3ac*, *H3K4me2 *and *H3K4me3*. To reproduce the clustering, see file *performHistoneClustering_Fig4CD.m *in the *examples *folder of the source code.

The ENCODE project was a large-scale community effort that sought to analyze 1% (30 Mb) of the human genome, through an array of experimental techniques that studied in detail the functions of selected DNA regions [13]. All assays performed were made accessible through the UCSC Encode Genome Browser through their web interface (Figure 4A) as well as through a DAS service [14].

One of the goals of ENCODE was to characterize histone modifications in normal human cell lines, e.g., GM06990 (lymphoblastoid) and HFL1 (lung fibroblast), and also in cancer cell lines, e.g., K562 (leukemia), HeLa (cervical carcinoma). Using ChIP-chip arrays [15], several H3 and H4 methylation and acetylation signals were measured, including *H3K4me1*, *H3K4me2*, *H3K4me3*, *H3K9me3*, *H3K27me3*, *H3K36me3*, *H3K79me3*, *H3ac*, *H4ac*. Taken together, these marks are a subset of what is known as the histone code. They act as a first-layer regulatory mechanism of gene expression, by inducing or repressing chromatin accessibility and recruitment of initiation factors [16].

We used histone modification data generated by ENCODE to exemplify how one can access data from DAS sources, and handle this experimental data to create new modes of visualization. Figure 4A shows how the UCSC Genome Browser exhibits information about histone data tracks, sorted by cell lines. The graphic in Figure 4B compares two specific positive histone marks, *H3K4me3 *and *H3ac*, measured in a normal (GM06990) and cancer cell line (K562), over chromosome 7. This side-by-side view of selected histone marks and selected cell lines facilitates the identification of ROIs to be further investigated. For example, looking at the graph we can outline that cancer cells have weaker positive marks when compared with normal cells, in regions located within bands q21.11 and q11.22 of chromosome 7. This is evidence of negatively modulated DNA, which may encode, for example, anti-tumorigenic functions. Other K562 ROIs are those that have gained positive marks, and therefore are likely to be more accessible for the DNA transcription machinery. Regions within bands p14.1 and p21.1 fall in this category as they have significant enrichment of *H3K4me3 *and *H3ac *modifications.

We also illustrate the potential of the API by creating an enriched histone data set that integrates information from multiple DAS sources. Figure 4C shows a heatmap of histone profiles in GM06990 cells for 5 marks, namely *H4ac*, *H3ac*, *H3K4me1*, *H3K4me2*, and *H3K4me3*. The data set for clustering was built by fetching ChIP-chip arrays from UCSC using the DASMiner. Then, the dataset of genomic regions with histone measurements was expanded by integrating two other DAS sources: the Vega/Havana Database [17] for retrieving gene annotation and the Genetic Association Disease database [18] for finding a cancer link. After retrieving these sources, a heatmap was generated where each column corresponds to a chromosome region that may be mapped to some gene, and this gene might be associated to some cancer type. Finally, the data was organized by hierarchical clustering using the Euclidean distance among histone modification profiles. This heatmap view provides an intuitive way to identify regions in the genome that share a similar histone modification pattern, and then to study these regions to characterize their function. In Figure 4D, we zoom in a selected a group of 39 regions with high signals for positive marks *H3ac*, *H3K4me2*, and *H3K4me3*. According to ENCODE findings, regions with this profile consist of very active transcribed DNA, and are usually associated with gene promoters. Within the group there are regions coding for genes TES, CAV1 and CAV2, which perform tumor suppressor activities. Also, from the GAD DAS annotation, we know that CAV1 and CAV2 are associated with prostate cancer.

### B) Creating and visualizing integrated molecular interaction data sets

**Figure 5:**
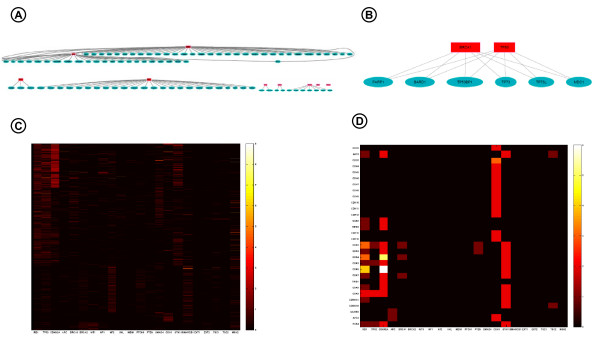
**Approaches for visualizing molecular interaction networks**. A) Protein network involving 22 established human TS and their interacting partners as found in PFAM DAS service [24], summing up 590 nodes and 771 edges (fragment is shown). TS are depicted in boxes, while other proteins are ellipses in the graph. Edges represent domain-domain interactions between two TS proteins, or most commonly between a TS and a non-TS protein. See *tumorSuppressorNetwork_Fig5AB.m *to run the example. B) Subgraph showing common domain-domain interactions for p53 and Brca1. C) Heatmap visualization of an integrated TS network created by fetching data from 11 DAS sources using DASMiner. D) Zooming in a group of 30 proteins in the heatmap. See *createTSConnectionHeatmap_Fig5CD.m *for a complete list of accessed DAS sources and to reproduce heatmaps C and D.

Another kind of molecular biology data available via DAS is protein interaction. The DASMI project [19] made available dozens of molecular interaction databases accessible via DAS protocol such as iPFAM, InterDom, Human Protein Reference Database, Bioverse, HomoMint and IntAct, to name just a few. We used this data to create an integrated model of a tumor suppressor (TS) network involving well-known human TS [20] and their interacting partners. Figure 5A illustrates a fragment of the TS network built using interactions reported in iPFAM. In this network, there is a connection between two proteins when their domains interact in 3D conformation. After representing this information in a network, we can interrogate it to extract knowledge regarding TS connectivity using graph algorithms. For example, we can find a subgraph of common interactors of p53 and Brca1, as depicted in Figure 5B. Both p53 and Brca1 participate in the DNA damage checkpoint during G1/S of cell cycle. They activate signalling pathways to carry out DNA repair and apoptosis in the cell, and these common interacting proteins are also participating in these processes. For example, Mdc1 is involved with double-stranded repair, while PARP1 acts in the base excision DNA repair [21].

For the other illustrative example, we built an integrated TS network using the information contained in 11 DAS sources, including PFAM and HPRD, and we visualized this data set using heatmaps. Figure 5C shows the TS network heatmap, where TS nodes are represented in columns, while non-TS are in rows. The color of a specific interaction is proportional to the number of hits supporting this interaction across different databases. Therefore, this heatmap exhibits how connected each TS is, and also allows assessing the reliability for a given TS/non-TS interaction. The visual inspection of this plot shows that Rb1, p53, Cdkn2a, Stk11, and Smarcb1 are among the most connected TS. Figure 5D provides a closer look over the heatmap, highlighting a group of 30 proteins and how they are linked to TS. For instance, we note that several cyclin-dependent kinases, i.e., cdk2, cdk3, cdk4, cdk5, cdk6, cdk7, cdk8, and cdk9, which are enzymes that control progression of the cell cycle, are usually found to be interacting with Rb1 and Cdkn2a, negative regulators of the cell cycle [21].

## Conclusion

The community of DAS sources provides an online collection of federated databases that covers most of the large repositories of molecular data. In this report we describe a novel tool, DASMiner, developed to explore these resources and to facilitate access to experimental data. DASMiner includes an adaptative user interface that can access any kind of DAS source, independent of the coordinate system or subset of commands implemented by the service. Together with the API, they can be used to deploy applications and create integrated views of biological systems using the information deposited in DAS repositories.

## Availability and Requirements

• Project name: DASMiner

• Project homepage: http://code.google.com/p/dasminer/

• Operating systems: Windows, Linux or Mac with Matlab installed.

• Programming language: Matlab. API examples were tested with Bioinformatics toolbox versions 2.5 and 3.2.

• License: The license is distributed under the GNU General Public License.

• Any restrictions to use by non-academics: none

## List of abbreviations

DAS: Distributed Annotation System; GUI: Graphical User Interface; REST: Representational State Transfer; ROI: Regions of Interest; TS: Tumor Suppressor(s).

## Authors' contributions

DFTV participated in the design, developed the tool, and drafted the manuscript; HFD and CA participated in the design; ATRV and JSA managed the project and improved the manuscript. All authors read and approved the final manuscript.
